# Obestatin induced recovery of myocardial dysfunction in type 1 diabetic rats: underlying mechanisms

**DOI:** 10.1186/1475-2840-11-129

**Published:** 2012-10-15

**Authors:** Manuela Aragno, Raffaella Mastrocola, Corrado Ghé, Elisa Arnoletti, Eleonora Bassino, Giuseppe Alloatti, Giampiero Muccioli

**Affiliations:** 1 Department of Experimental Medicine and Oncology, University of Turin, Corso Raffaello 30, Turin, 10125, Italy; 2 Department of Drug and Science Technology, University of Turin, Via Pietro Giuria 13, Turin, 10125, Italy; 3 Department of Life Sciences and Systems Biology, University of Turin, Via Accademia Albertina 13, Turin, 10123, Italy

**Keywords:** Obestatin, Diabetes, Cardiac dysfunction, Oxidative unbalance, Pro-fibrogenic and inflammatory factors, Cell survival kinases

## Abstract

**Background:**

The aim of this study was to investigate whether obestatin (OB), a peptide mediator encoded by the ghrelin gene exerting a protective effect in ischemic reperfused heart, is able to reduce cardiac dysfunctions in adult diabetic rats.

**Methods:**

Diabetes was induced by STZ injection (50 mg/kg) in Wistar rats (DM). OB was administered (25 μg/kg) twice a day for 6 weeks. Non-diabetic (ND) rats and DM rats were distributed into four groups: untreated ND, OB-treated ND, untreated DM, OB-treated DM. Cardiac contractility and ß-adrenergic response were studied on isolated papillary muscles. Phosphorylation of AMPK, Akt, ERK1/2 and GSK3ß as well ß-1 adrenoreceptors levels were detected by western blot, while α-MHC was measured by RT-PCR.

**Results:**

OB preserved papillary muscle contractility (85 vs 27% of ND), ß-adrenergic response (103 vs 65% of ND), as well ß1-adrenoreceptors and α-MHC levels in diabetic myocardial tissue. Moreover, OB up-regulated the survival kinases Akt and ERK1/2, and enhanced AMPK and GSK3ß phosphorylation. OB corrected oxidative unbalance, reduced pro-inflammatory cytokine TNF-α plasma levels, NFkB translocation and pro-fibrogenic factors expression in diabetic myocardium.

**Conclusions:**

OB displays a significant beneficial effect against the alterations of contractility and ß-adrenergic response in the heart of STZ-treated diabetic rats, which was mainly associated with the ability of OB to up-regulate the transcription of ß1-adrenergic receptors and α-MHC; this protective effect was accompanied by the ability to restore oxidative balance and to promote phosphorylation/modulation of AMPK and pro-survival kinases such as Akt, ERK1/2 and GSK3ß.

## Introduction

Obestatin (OB) is a newly discovered peptide encoded by the ghrelin gene together with acylated (AG) and unacylated ghrelin (UAG), produced in the oxyntic mucosa of the stomach [1-3]. It has been initially suggested that OB behaves as a physiological opponent to ghrelin, through interaction with the orphan G-protein-coupled receptor 39 (GPR39) [4]. However, these findings have been questioned lately and a number of studies have failed to confirm the anorexigenic effect of obestatin [5-7] and its agonist properties on GPR39 [8,9]. Therefore, to date, some biological actions of OB seem to be controversial and its specific receptor remains unknown. Recent data suggest a relevant role of peptides encoded by the ghrelin gene in glucose balance and their implications for diabetes. Although the direct protective effects exerted by ghrelin gene-derived peptides on β-cells and pancreatic islets has been demonstrated in streptozotocin (STZ)-treated neonatal rats [10-13], to our knowledge, limited data exist regarding their ability to protect the myocardial tissue in adult diabetic animals, in which the ability to induce β-cell regeneration is lost.

OB plays a significant role in cardiac function in humans, both under physiological and pathological conditions. In particular, higher saliva OB levels were found in overweight patients with ischemic heart disease compared with healthy controls [14], whereas they are significantly decreased in serum of subjects with type 2 diabetes mellitus [1] and obesity [15]. Increased OB levels and altered ghrelin to OB ratio are present in chronic heart failure (CHF) patients with cachexia [16], as well in spontaneously hypertensive rats [17]. OB improves myocardial function and reduces cell death and apoptosis of cardiomyocytes after ischemia/reperfusion (I/R) in isolated rat heart [18]. These effects are probably mediated by OB receptors present on cardiac cells and by activation of signaling pathways included in the reperfusion injury salvage kinases (RISK), such as phosphoinositide 3-kinase (PI3K), protein kinase C (PKC) and extracellular signal regulated kinases (ERK) 1/2 [18]. In type 1 diabetes, hyperglycemia is the active element leading to cardiac dysfunctions. Hyperglycemia promotes the production of reactive oxygen species (ROS) and contributes to cardiac fibrosis and contractile dysfunctions [19-23]. The protective effects exerted by OB against myocardial dysfunctions in ischemic-reperfused heart led us to investigate whether OB may rescue myocardial contractility and beta adrenergic response in the heart of STZ treated diabetic rats. The main results obtained in this study suggest that OB displayed a beneficial effect against the reduction of contractility and β-adrenergic response, as well of α-myosin heavy chain (MHC) and β-1 adrenoreceptors expression in the heart of STZ-treated diabetic rats, and that the protective effect is probably related to the ability of OB to restore oxidative balance and to promote phosphorylation/modulation of AMPK and pro-survival kinases such as Akt, ERK1/2 and glycogen synthase kinase (GSK) 3β.

## Materials and methods

### Cell culture

H9c2 cardiomyoblasts, an embryonic rat heart-derived cell line, were obtained from the Istituto Zooprofilattico Sperimentale della Lombardia e dell’Emilia (Brescia, Italy) and cultured in 25 cm^2^ flasks with Dulbecco’s Modified Eagle Medium (DMEM) containing 10% foetal calf serum (FCS) at 37°C in a humidified atmosphere with 95% O_2_ and 5% CO_2_. Cells were starved for 24 hours in DMEM with 1% FCS and then treated with 20 mM metformin (Sigma-Aldrich, Milan, Italy), as positive control for AMPK phosphorylation, or 50 nM OB (rat obestatin (1–23), produced by conventional solid-phase synthesis and purified (97%) by reverse-phase HPLC by NeoMPS, Strasbourg, France). H9c2 were then scraped, centrifugated (1500 rpm, 5 minutes, 4°C) and lysed for total protein extraction.

### Animals

Male Wistar rats (Harlan-Laboratories, Udine, Italy) weighing 200–220 g were provided with Piccioni pellet diet (no. 48, Gessate Milanese, Italy) and water *ad libitum*. The animals were cared for in compliance with the Local Ethical Committee and Italian Ministry of Health Guidelines (no. 86/609/EEC) and with the *Principles of Laboratory AnimalCare* (NIH no. 85–23, revised 1985). The scientific project was supervised and approved by the Italian Ministry of Health, Rome, and by the ethical committee of the University of Torino.

### Animals acute treatment

The effects of an acute treatment with OB or metformin were tested in a group of fifteen rats. Five rats received a daily i.p. injection of metformin (250 mg/kg/day) for three consecutive days, as indicated by Zhou *et al.*[24]. A second group of five rats received a single dose of OB by i.p. injection 1 hour before killing, following the same procedures described by Kola *et al.*[25] for ghrelin treatment. Five control rats received intraperitoneal injections of saline. All rats were anesthetized with 90 mg /kg of Zoletil 100 i.p. (Virbac, Carros, France) and killed by aortic exsanguinations. Hearts were removed and left ventricle was excised and rapidly homogenized to obtain protein extracts for western blotting analyses.

### Animals chronic treatment

In a group of rats basal glucose level was measured by saphenous vein puncture with the Accu-Check Compact kit (Roche Diagnostics Gmbh, Mannheim, Germany). Hyperglycemia was induced in a group of rats through a single injection of streptozotocin (STZ) (50 mg/kg b.wt) in the tail vein (diabetic, DM). A group of rats received only the vehicle (non-diabetic, ND). Three days later glucose was measured and only rats with blood glucose levels above 200 mg/dL entered the experimental protocols. ND and DM rats were distributed into four groups: untreated ND (*n =* 18) (ND-CTRL), obestatin-treated ND (*n =* 10) (ND-OB), untreated DM (*n =* 20) (DM-CTRL); obestatin-treated DM (*n =* 18) (DM-OB). OB was administered subcutaneously twice a day for 6 weeks, at the final dose of 25 μg/kg/5 ml.

Two days before the sacrifice, after a fasting period of 12 h, a 50% glucose solution at the dose of 3 g/kg was orally administered to the rats. Glucose levels were tested 0, 15, 30, 45, 60, 90, 120 and 240 min after glucose loading by saphenous vein puncture.

Rats were sacrificed after 6 weeks. All rats were anesthetized with Zoletil 100 i.p. and killed by aortic exsanguinations. Blood was collected and the plasma isolated and placed at −80°C. Glucose level was immediately measured. The heart was isolated, weighed and portions of left ventricle were immediately homogenized to obtain different tissue extracts. To evaluate papillary muscles contractility and β-adrenergic response, papillary muscles were isolated from left ventricle, as described below.

### Plasma detection

Triglyceride (TG), total cholesterol (TC), high-density-lipoprotein (HDL) and low density-lipoprotein (LDL) were determined in plasma by standard enzymatic procedures using reagents kits (Hospitex Diagnostics, Florence, Italy). Plasma insulin was measured by an ultrasensitive insulin enzyme-linked immunosorbent assay kit from DRG Diagnostics (Marburg, Germany). The content of TNF-α was determined by using a rat TNF-α ELISA kit (Diaclone, Besancon, France) following the manufacturer’s guidelines. Each sample was tested in duplicate and averaged. Final results were calculated from the kit standards and expressed as picograms of TNF-α per milliliter.

### Protein extraction

For cytosolic and nuclear proteins extraction, left ventricles were homogenized at 10% (w/v) using a Potter Elvehjem homogenizer (Wheaton, NJ, USA) in a homogenization buffer [20 mM HEPES, pH 7.9, 1 mM MgCl_2_, 0.5 mM EDTA, 1 mM EGTA, 1 mM dithiothreitol (DTT), 0.5 mM phenylmethylsulphonyl fluoride (PMSF), 5 μg/ml aprotinin, and 2.5 μg/ml leupeptin]. Homogenates were cleared by centrifugation at 1,000 g for 5 min at 4°C. Supernatants were removed and centrifuged at 15,000 g at 4°C for 40 min to obtain the cytosolic fraction. The pelleted nuclei were resuspended in an extraction buffer [20 mM HEPES, pH 7.9, 1.5 mM MgCl_2_, 0.2 mM EDTA, 20% (w/v) glycerol, 1 mM EGTA, 1 mM DTT, 0.5 mM PMSF, 5 μg/ml aprotinin, and 2.5 μg/ml leupeptin]. The suspensions were incubated on ice for 30 min for high-salt extraction followed by centrifugation at 15,000 g for 20 min at 4°C. The resulting supernatants containing nuclear proteins were carefully removed and stored.

Total extracts were obtained both from 10% (w/v) left heart ventricle homogenates and cell lysates in RIPA buffer [0.5% Nonidet P-40, 0.5% sodium deoxycholate, 0.1% SDS, 10 mmol/l EDTA, and protease inhibitors]. After 40 minutes of incubation in ice, samples were cleared by centrifugation at 15,000 g at 4°C for 40 min. Supernatants were removed and stored. Protein content was determined using the Bradford assay. Protein extracts were stored at −80°C until use.

### Oxidative biochemical parameters

The levels of reactive oxygen species (ROS) were measured on total extracts using the probe 2’,7’-dichlorofluorescin diacetate (DCFH-DA) and measured fluorimetrically [26]. Reduced and oxidized glutathione content was evaluated in cytosolic fractions following Owens’s method [27]. The difference between total glutathione and reduced GSH content represents the GSSG content (expressed as μg/mg prot.); the ratio between GSSG content and GSH is considered a good parameter of antioxidant status. Hydroxynonenal (HNE) concentration was also determined on cytosol fractions. After extraction, an aliquot of cytosol was injected into an HPLC (Waters Associated, Milford, MA, USA) Symmetry C_18_ column (5 mm, 3.9x150 mm). The mobile phase was acetonitrile:bidistilled water (42%, v/v). The HNE concentration was calculated by comparison with a standard solution of HNE of known concentration.

### Western blot

NFkB-p65 was detected on cytosol and on nuclear extracts; AMPK, pAMPK, Akt, pAkt, ERK1/2, pERK1/2, pGSK3β, β1-adrenoreceptor and β-actin were detected on total extracts. Equal amounts of proteins (60 μg) were separated on 7.5% SDS-polyacrylamide gels, then blotted on nitrocellulose membranes (Amersham Biosciences, Braunschweig, Germany). The membranes were incubated overnight with primary antibodies and reacted with appropriated peroxidase-labeled secondary antibodies (Biorad). Immunoreactive proteins were detected through the chemiluminescence assay (ECL, Amersham) and subsequent exposure to film.

Specific bands were quantified by densitometry using an analytic software (Bio-Rad, Multi-Analyst, Munchen, Germany) and the net intensity of phosphorylated proteins was normalized for the intensity of the corresponding total protein. β-actin was used as internal loading control for pGSK3β and cytosolic NFkB, while lamin A was used for nuclear NFkB.

### Semiquantitative RT-PCR

Total RNA was extracted from left ventricle using TriPure Isolation Reagent (Roche Diagnostics). Gene transcripts for α- and β-MHC, β1-adrenoreceptor, CTGF, TGFβ1 and GADPH were quantified by PCR. All experiments were performed on at least three independent cDNA preparations. PCR products were electrophoresed on 2% agarose gels and amplification products were stained with GelStar nucleic acid gel stain (FMC BioProducts, Rockland, ME, USA). Gels were photographed and analyzed with Kodak 1D Image Analysis software. The primer sequences were: α-MHC forward 5’ AGCCTCTCATCTCGCATCTC 3’; reverse 5’ GGACCACCCATC TCACTTT 3’, β-MHC forward 5’ ACCGCTGAGACAGAGAATGG 3’; reverse 5’ GGGTTGGCTTGGATGATTT 3’, β1-adrenoreceptor forward 5’ GCCGATCTGG TCATGGGA 3’; reverse 5’ GTTGTAGCAGCGGCGCG 3’, CTGF forward 5’ CAAGGACCG CACGTGGTT 3’; reverse 5’ CCCTAGCTGCCTACCGACTGGAAGACAC 3’**,** TGFβ1 forward 5’ CTGCTGGCAA TAGCTTC CTA – 3’; reverse 5’ CGAGCCTTAGTTTGGACAGGAT 3’; GAPDH forward 5’ AGATCCACAACGGATACATT 3’; reverse 5’ TCCCTCAAGATTGTCAGCAA 3’.

### Papillary muscle and contractility determination

Papillary muscles isolated from the left ventricle were superfused with oxygenated (100% O_2_) Tyrode solution containing (in mM): 154 NaCl, 4 KCl, 2 CaCl_2,_1 MgCl_2_, 5.5 glucose, 5 N-(2-hydroxyethyl)-piperazineN’-ethanesufonic acid (HEPES); pH 7.4 adjusted with NaOH) warmed to 37°C, and driven at constant frequency (120 beats/min). Isometric twitches were evaluated by a 60–2997 Harvard transducer, visualized on a Tektronix 2211 digital storage oscilloscope and acquired and recorded in a Power Mac computer, using the Labview Software (National Instruments Corp., Texas, USA). To study the responsiveness to β-adrenergic stimulation, after 20–30 min equilibration, papillary muscles obtained from the different groups of animals were treated with isoproterenol (Iso, 1 μM). Preliminary studies showed that 1 μM was the minimal saturating concentration of Iso.

### Statistical analysis

All values were expressed as means ± SD, and were analyzed by one-way analysis of variance (ANOVA) followed by Bonferroni’s multiple comparison test or by Newman-Keuls multiple range test, when appropriate. *P* < 0.05 was considered statistically significant.

## Results

### Obestatin restores papillary muscle contractility in diabetic rats

To test whether OB is able to protect against the alterations of cardiac function induced by diabetic condition, contractile force developed by electrically-driven papillary muscles was evaluated both in basal conditions and after stimulation with Iso (1 μM) to compare basal cardiac contractility and responsiveness to β-adrenergic stimulation among the different groups of rats (Table 1, panel A and B). As expected [28], basal contractility (panel A) was significantly weaker in papillary muscles from untreated diabetic rats compared with untreated non-diabetic muscles; this was evident not only for the maximal developed mechanical tension (T_max_), but also for the maximum rate of rise (+dT/dt_max_) and the maximum rate of fall of developed mechanical tension (−dT/dt_max_) (in all the cases, p < 0.001), while no significant difference was found for the time to peak of mechanical tension (TPT) and duration of contraction. Treatment with OB significantly limited the reduction of contractility observed in untreated diabetic rats (p < 0.001). However, OB treatment in non-diabetic rats did not affect *per se* contractile properties of papillary muscles. Moreover, papillary muscles from untreated diabetic rats showed a reduced inotropic response to β-adrenergic stimulation, which was 55% stronger in papillary muscles of untreated non-diabetic rats (p < 0.01: Table 1, panel B). This difference was also evident for + dT/dt_max_, -dT/dt_max_ and TPT. Treatment with OB significantly rescued the responsiveness of papillary muscles to β-adrenergic stimulation (p < 0.01 for T_max_, +dT/dt_max_, -dT/dt_max_ and TPT) in diabetic rats, while did not affect the response to Iso in non-diabetic rat. OB treatment did not modify heart weight and heart to body weight ratio in non-diabetic rats, while diabetic rats showed a marked decrease in heart weight compared to non-diabetics ones (Table 2). OB treatment in diabetic rats only led to a slight, not significant reduction in heart to body weight ratio compared to untreated diabetic animals. Thus, OB-treated non-diabetic rats showed a decrease (−20%) of body weight compared to untreated non-diabetic rats. OB treatment of diabetic rats did not further reduce body weight in comparison to untreated diabetic rats (about −44% *vs* CTRL) (Table 2).

**Table 1:** **A. Basal values forcardiac contractility in papillaryheart muscles of untreatedand OB-treated non-diabetic ratsand in untreated andOB-treated diabetic rats**

**A**				
	**NON-DIABETIC**		**DIABETIC**	
	**CTRL (*n* = 6)**	**OB** (***n* = 5)**	**CTRL (*n* = 6)**	**OB (*n* = 5)**
**T**_**max**_ (mN/mm^2^)	50.3 ± 7.7	42.8 ± 8.4	13.4 ± 6.9^***^	60.2 ± 20.5^†††^
**+ dT/dt**_**max**_ (mN/s)	15265 ± 1759	14043 ± 1527	5420 ± 847^***^	14688 ± 2165^†††^
**- dT/dt**_**max**_ (mN/s)	7297 ± 874	6785 ± 645	4056 ± 953^***^	6333 ± 1135^††^
**TPT** (ms)	133.3 ± 14.2	114.5 ± 13.6	111.9 ± 31.4	149.6 ± 9.0
**Duration** (ms)	379.5 ±48.7	353.5 ± 20.2	361.7 ± 75.4	407.6 ± 26.2
**B***Isoproterenol (1 μM)*				
	**NON-DIABETIC**		**DIABETIC**	
	**CTRL (*n* = 6)**	**OB (*n* = 5)**	**CTRL (*n* = 6)**	**OB (*n* = 5)**
**T**_**max**_**%**	189.7 ± 27.8	197.3 ± 48.0	123.8 ± 18.0^**^	183.2 ± 22.7^††^
**+ dT/dt**_**max**_**%**	192.7 ± 40.6	205.0 ± 39.2	127.3 ± 16.8^**^	190.0 ± 31.0^††^
**- dT/dt**_**max**_**%**	250.3 ± 28.1	227.6 ± 28.4	126.0 ± 14.2^***^	187.6 ± 31.1^††^
**TPT%**	85.0 ± 2.4	86.6 ± 5.9	97.8 ± 4.1^***^	90.8 ± 3.3^††^
**Duration%**	83.3 ± 3.4	85.5 ± 10.6	95.6 ± 5.8	90.0 ± 6.8

B. Effect of Iso (1 μM) on cardiac contractility. Value expressed as % variation respect to control (before Iso). Papillary muscles did not significantly differ in size among the different groups (diameter: 0.78 ± 0.08, 0.81 ± 0.12, 1.23 ± 0.15 and 1.18 ± 0.09 mm, respectively).

Values are expressed as means ± SD. Statistical significance: ** *P* < 0.01*vs* ND-CTRL; *** *P* < 0.001 *vs* ND-CTRL; †† *P* < 0.01 *vs* DM-CTRL; ††† *P* < 0.001 *vs* DM-CTRL.

**Table 2:** **Body weight, heart weight,heart/body weight ratio, glucose,insulin level and lipidprofile in plasma ofuntreated and OB-treated non-diabeticrats and in untreatedand OB-treated diabetic rats,evaluated at the endof the protocol time(6 weeks)**

	**NON-DIABETIC**		**DIABETIC**	
	**CTRL (*n* = 12)**	**OB (*n* = 5)**	**CTRL (*n* = 14)**	**OB (*n* = 13)**
**BODY WEIGHT** (g)	425 ± 14	344 ± 12^*^	258 ± 26^**^	233 ± 27^**^
**HEART WEIGHT** (g)	0.89 ± 0.06	0.88 ± 0.08	0.57 ± 0.05^***^	0.52 ± 0.05^***^
**HEART/BODY WEIGHT** (g/mg)	2.63 ± 0.17	2.57 ± 0.07	3.20 ± 0.22^**^	3.09 ± 0.21^*^
**GLUCOSE** (mg/dL)	104 ± 18	99 ± 11	388 ± 27^***^	372 ± 120^***^
**INSULIN** (μg/L)	0.42 ± 0.02	0.49 ± 0.05	0.18 ± 0.04^***^	0.17 ± 0.03^***^
**TG** (mg/dL)	100.95 ± 6.69	102.26 ± 9.11	356.68 ± 57.03^***^	255.09 ± 21.84^**†^
**LDL** (mg/dL)	56.78 ± 7.46	59.10 ± 9.22	169.19 ± 14.46^***^	112.41 ± 11.98^**†^
**HDL** (mg/dL)	55.99 ± 5.50	54.26 ± 2.69	25.14 ± 3.38^***^	40.89 ± 8.67^*†^
**TC** (mg/dL)	92.58 ± 3.29	92.91 ± 9.57	123.11 ± 16.68^*^	104.49 ± 5.41^†^

Values are expressed as means ± SD. Statistical significance: * *P* < 0.05 *vs* ND-CTRL; ** *P* < 0.01 *vs* ND-CTRL; *** *P* < 0.005 *vs* ND-CTRL; † *P* < 0.05 *vs* DM-CTRL.

### Obestatin promotes β1-adrenoreceptors and α-MHC expression recovery in diabetic myocardial tissue

**Figure 1:**
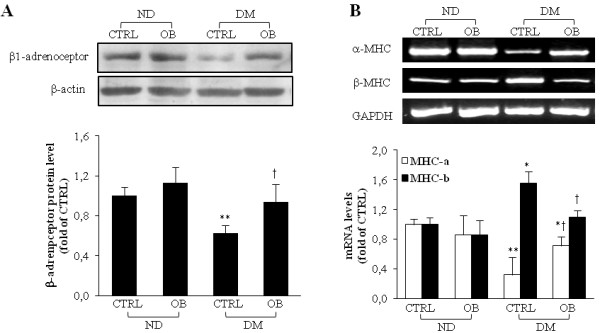
**Representative WB analyses ofβ1-adrenoreceptor (A) and semiquantitativeRT-PCR analyses of α-MHCand β-MHC (B) expressionsin left ventricular tissueRNA extracts from non-diabetic(ND) and diabetic rats(DM), treated or notwith OB.** Quantitative analyses are reported in the histograms, which represent the ratio of net intensity of bands with GAPDH. Data are expressed as percentage variations from the ND-CTRL value. Data are means ± SD of 8–10 rats per group. Statistical significance: * *P* < 0.05 *vs* ND-CTRL; ** *P* < 0.01 *vs* ND-CTRL; † *P* < 0.05 DM-CTRL.

The reduced response to Iso stimulation observed in the heart of diabetic rats has been related to a limited expression of β1-adrenoreceptors [29,30]. While β1-adrenoreceptors protein expression showed a marked reduction in ventricular tissue of diabetic rats, a significant recovery was observed in diabetic rats treated with OB (p < 0.05 *vs* diabetic rats, Figure 1, panel A).

The changes reported in diabetic myocardial tissue include the switch from α to β myosin isoform chains, the accumulation of fibronectin and of types I and III collagen matrix [29]. The expression of α and β isoforms of the MHC protein in the left ventricle of all groups obtained by PCR analysis has been reported in Figure 1, panel B. In untreated diabetic rats we observed a decrease in α-MHC and an increase in β-MHC isoform. OB treatment in diabetic rats partially rescued the α/β MHC ratio with respect to untreated diabetic rats. OB treated non-diabetic rats did not showed any modification of α/β MHC ratio. On the basis of obtained data, we looked for molecular mechanisms underlying the protective effects of OB against contractile impairments induced by diabetic state, by studying OB effects on oxidative stress, inflammatory response, on specific pro-survival kinases and pro-fibrogenic factors.

### Obestatin corrects oxidative unbalance in diabetic heart

The levels of ROS as well as HNE, an end-product of lipid peroxidation, evaluated in cytosolic fraction of left ventricular tissue, increased significantly in untreated diabetic rats (Table 3). When OB was given to diabetic rats, ROS and HNE levels were drastically reduced if compared to untreated diabetic rats, being carried back to the values recorded in untreated non-diabetic rats. Moreover, the significant increase of GSSG/GSH ratio observed in diabetic rats further confirmed the occurrence of oxidative stress. In OB treated diabetic rats, this ratio was partially decreased towards values recorded in non-diabetic rats, suggesting a recovery of the GSH content (Table 3). OB treatment of non-diabetic rats did not showed *per se* any significant effects on oxidative parameters in comparison with untreated non-diabetic rats.

**Table 3:** **Oxidative stress parameters evaluatedin cardiac tissue ofcontrol, OB, STZ andSTZ-treated OB rats**

	**NON-DIABETIC**		**DIABETIC**	
	**CTRL (*n* = 12)**	**OB (*n* = 6)**	**CTRL (*n* = 14)**	**OB (*n* = 14)**
**ROS** (U.F./mg prot)	106.67 ± 2.08	106.54 ± 15.44	165.67 ± 15.40^*^	116.43 ± 18.02^†^
**GSSG/GSH (%)**	1.60 ± 1.00	1.64 ± 0.40	8.91 ± 2.60^***^	4.43 ± 3.20^†^
**HNE (μM)**	0.80 ± 0.08	0.83 ± 0.08	1.79 ± 0.12^**^	1.02 ± 0.17^††^

Values are expressed as means ± SD. Statistical significance: * *P* < 0.05 *vs* ND-CTRL; ** *P* < 0.01 *vs* ND-CTRL; *** *P* < 0.005 *vs* ND-CTRL; † *P* < 0.05 *vs* DM-CTRL; †† *P* < 0.01 *vs* DM-CTRL.

### Obestatin reduces pro-inflammatory cytokine TNF-α plasma levels and NFkB translocation in the diabetic myocardium

**Figure 2:**
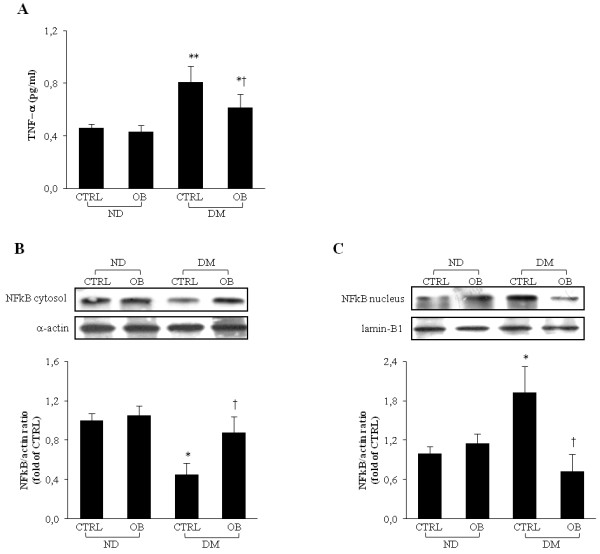
**Representative TNFα plasma concentrationdetermined by ELISA (A).** Representative WB analyses of NFkB-p65 in cytosol (**B**) and in nucleus (**C**) obtained from left ventricular tissue from non-diabetic (ND) and diabetic rats (DM), treated or not with OB. Quantitative analyses are reported in the histograms, which represent the net intensity ratio of bands with α-actin or lamin-B1. Data are expressed as % variations from the ND-CTRL value. Data are means ± SD of 8–10 rats per group. Statistical significance: * *P* < 0.05 *vs* ND-CTRL; ** *P* < 0.01 *vs* ND-CTRL; † *P* < 0.05 *vs* DM-CTRL.

High levels of cytokines have been found in diabetic patients and several studies report the stimulatory effects of high glucose on cytokines production and on NFkB-dependent signaling activation. OB treatment in diabetic rats determined a reduction of both the pro-inflammatory cytokine TNF-α level (Figure 2, panel A) and the activation of NFkB translocation in the nucleus (Figure 2, panel B and C).

### Obestatin up-regulates survival kinases Akt and ERK1/2 in diabetic left ventricular tissue

**Figure 3:**
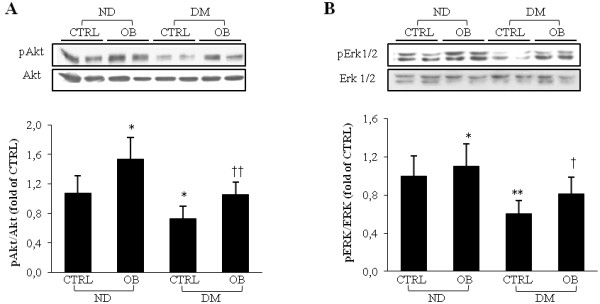
**Representative WB analyses ofpAkt (A) and pERK1/2(B) on left ventricletotal extracts from non-diabetic(ND) and diabetic rats(DM), treated or notwith OB.** Quantitative analyses are reported in the histograms, which represent the net intensity ratio of bands with total Akt or ERK1/2. Data are expressed as % variations from the ND-CTRL value. Data are means ± SD of 8–10 rats per group. Statistical significance: * *P* < 0.05 *vs* ND-CTRL; ** *P* < 0.01 *vs* ND-CTRL; † *P* < 0.05 *vs* DM-CTRL; †† *P* < 0.01 *vs* DM-CTRL.

We observed a significant reduction of Akt and ERK1/2 phosphorylation levels (Figure 3, panel A and B, respectively) in untreated diabetic rats. OB treatment significantly enhanced the phosphorylation levels of both these kinases in non-diabetic rats and rescued their levels in diabetic rats.

### Obestatin decreases pro-fibrogenic factors expression in diabetic heart

**Figure 4:**
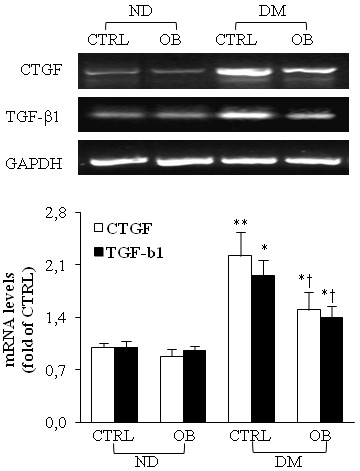
**Representative semiquantitative RT-PCR analysesof CTGF and TGF-β1in left ventricle RNAextracts from non-diabetic (ND)and diabetic rats (DM),treated or not withOB.** Quantitative analyses are reported in the histogram, which represents the ratio of net intensity of bands with GAPDH. Data are expressed as % variations from the ND-CTRL value. Data are means ± SD of 8–10 rats per group. Statistical significance: * *P* < 0.05 *vs* ND-CTRL; ** *P* < 0.01 *vs* ND-CTRL; † *P* < 0.05 DM-CTRL.

In our study, untreated diabetic rats showed a marked activation of the expression of CTGF and TGF-β, which represent two important pro-fibrogenic factors, responsible for up-regulated extracellular matrix production. OB caused a significant reduction in CTGF and TGF-β1 expression respect to that observed in untreated diabetic rats, as reported in Figure 4. OB treatment in non-diabetic rats did not exert any modification in comparison with untreated non-diabetic rats.

### Obestatin enhances AMPK and GSK3β phosphorylation

**Figure 5:**
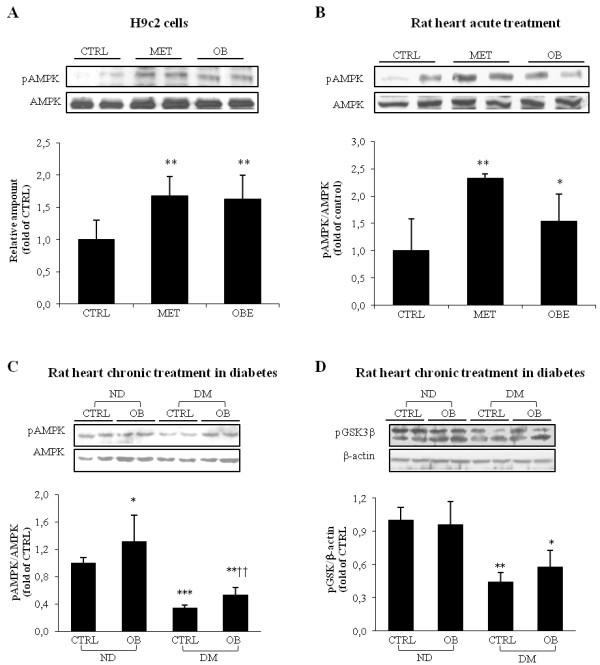
**Representative WB analyses ofpAMPK in H9c2 cellstreated with metformin orobestatin (A), in leftventricle of rat subjectedto acute treatment withmetformin or OB (B)and in left ventricleof non-diabetic (ND) anddiabetic rats (DM), treatedor not with OBfor 6 weeks (C).** Representative WB analyses of pGSK3β (**D**) in left ventricle of non-diabetic (ND) and diabetic rats (DM), treated or not with OB for 6 weeks. Quantitative analyses are reported in the histograms, which represent the net intensity ratio of bands with total AMPK or β-actin. Data are expressed as percentage variations from the ND-CTRL value. Data are means ± SD of 8–10 rats per group. Statistical significance: * *P* < 0.05 *vs* ND-CTRL; ** *P* < 0.01 *vs* ND-CTRL; *** *P* < 0.005 *vs* ND-CTRL; † *P* < 0.05 *vs* DM-CTRL; †† *P* < 0.01 *vs* DM-CTRL.

In preliminary experiments to verify whether OB is able to directly enhance AMPK phosphorylation in H9c2 myocardiocytes (Figure 5, panel A) and in acutely OB treated rats (Figure 5, panel B), we observed that the effect of OB was comparable to those induced by metformin, a well-known AMPK activator [31]. In accordance with these results, we observed an up-regulation of AMPK in OB non-diabetic rats (Figure 5, panel C). In addition, OB treatment partially rescued the pAMPK level in diabetic rats, which showed a marked reduction of AMPK activity. In addition, the levels of GSK3β, a substrate of pAkt and pAMPK activity, decreased in untreated diabetic rats (Figure 5, panel D). OB treatment in diabetic rats determined a partial recovery of pGSK3β levels. Taken together, our results suggested that the protective effect of OB against contractile dysfunction induced by chronic hyperglycemia is, at least in part, due to its ability to rescue AMPK and GSK3β phosphorylation.

### Obestatin decreases hyperlipemia but not hyperglycemia in diabetic rats

**Figure 6:**
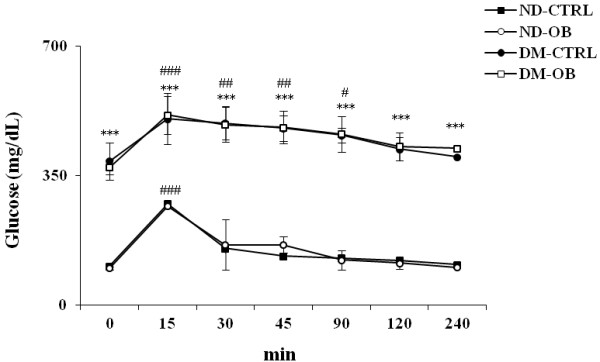
**Blood glucose tolerance testin non-diabetic (ND) anddiabetic rats (DM), treatedor not with OB.** Concentration time-profile after glucose load given *per os* as described in Materials and Methods. Data are mean ± SD for six-ten animals per group. Statistical significance: *** *P* < 0.001*vs* ND-CTRL; ### *P* < 0.001 *vs* time 0.

Glycemia and insulin levels, measured after 6 weeks from STZ-injection, reflected the hyperglycemic state caused by the significant reduction of insulin. OB treatment did not determine any modification of glycemia as well as insulin plasma level in both diabetic and non-diabetic rats (Table 2). In order to find out the effect of OB on glucose sensitivity, we performed an oral glucose tolerance test at 6 weeks after STZ injection. Figure 6 shows the effects of oral glucose loading (3 g/kg) in all groups. In untreated non-diabetic rats and in OB treated non-diabetic rats, glucose loading transiently increased plasma glucose levels at time 15 min, with respect to baseline. In these groups, glycemia returned to normal values 30 min after glucose loading. The high glucose level already observed in diabetic rats at time 0, was further slightly increased 15 min after glucose loading and remained significantly higher for up to 240 min. The time courses describing plasma glucose trajectory in untreated and OB treated diabetic rats showed a not significant difference, indicating that OB treatment was not able to reduce the high glucose levels found in untreated diabetic rats.

OB given to non-diabetic rats did not modify lipid profile with respect to untreated non-diabetic rats. Untreated diabetic rats showed a significant increase in plasma lipid content (triglycerides, LDL, total cholesterol) *vs* both untreated non-diabetic rats and OB treated non-diabetic rats. OB treatment in diabetic rats partially recovered lipid levels in comparison with untreated diabetic rats (Table 2). The decrease of HDL level observed in diabetic rats was partially rescued in OB treated diabetic rats. Thus, OB treatment, in non-diabetic and diabetic rats, did not exert any alteration on glycidic profile, while OB significantly protect against changes of lipid plasma levels of diabetic rats.

## Discussion

The major new findings in this study are that: 1) OB displayed a beneficial effect against the alterations of contractility and β-adrenergic response in the heart of STZ-treated diabetic rats; 2) the protective effect is related to the ability of OB to restore oxidative balance and to promote phosphorylation/modulation of AMPK and pro-survival kinases such as Akt, ERK1/2 and GSK3β. To our knowledge, this is the first report showing that OB is able to exert a protective action in myocardial tissue affected by experimentally induced diabetes in adult animals.

OB is a newly discovered peptide, which derives from the ghrelin peptide precursor pre-pro-ghrelin [1,2,4]. Although it has been suggested that both OB and ghrelin participate in a complex regulatory system [32], the intracellular pathways activated by OB and the role it plays in physiological and in pathophysiological conditions are largely unknown. Several studies have already indicated that, in common with ghrelin and other growth hormone secretagogues, OB protects cardiac muscle against I/R injury [33]. Moreover, it has been shown that ghrelin reduces oxidative stress and inhibits the production of reactive oxygen species [34,35], biological events that have been implicated in various pathologies, including hypertension [36], cardiac ischemia [37] and myocardial fibrosis [19,20,23,29]. A similar protective effect has been also reported in primary cardiomyocytes, as well as in H9c2 cardiomyoblastic cell line and endothelial cells, in which both ghrelin [21] and OB [18] inhibit apoptosis induced by doxorubicin or I/R, by activating ERK1/2, PKC and PI3K/Akt dependent mechanisms. Several evidences indicate that ghrelin gene-derived peptides prevent the development of diabetes in STZ-treated newborn rats. Indeed, both ghrelin and OB promote regeneration of β-cells and improve glucose metabolism in STZ-treated newborn rats, granting a therapeutic potential in medical conditions associated with impaired β-cell function [10-13]. Moreover, a recent paper showed that ghrelin ameliorated the reductions in motor and sensory nerve conduction velocities and reduced oxidative stress in STZ-treated mice [38], suggesting that ghrelin gene-derived peptides could also be able to protect against dysfunction in experimentally-induced diabetes in adult animals, in which it is likely these peptides have lost their ability to induce β-cell regeneration. In addition, ghrelin improves both the metabolic functions and the disturbed energy metabolism in the cardiac muscle of obese diabetic rats [39].

Among all diabetic complications, cardiovascular disease continues to be the primary cause of morbidity and mortality. In agreement with other studies [16,40], we previously reported that diabetic cardiomyopathy induced by chronic hyperglycemia is characterized by myocyte loss and myocardial fibrosis, which lead to decreased elasticity and impaired contractile function [29]. Moreover, the enhanced oxidative stress reduced peak contractile amplitude and maximal velocity of contraction and relaxation under basal condition, as well as the β-adrenergic response in diabetic myocardial tissue [29,30].

This study demonstrates that OB exerts a protective effect against the derangement of contractility observed in papillary muscles from diabetic rats (−65% of mechanical tension *vs* control). The beneficial effect of OB could be due to its ability to counteract the switch of cardiac myosin heavy chain gene expression from the α- to the β-MHC isoform, and the increase of pro-fibrogenic growth factors, such as TGFβ1 and CTGF, observed in diabetic myocardial tissue. In addition, OB was also able to restore the β-adrenergic response by promoting recovery of β1-adrenoreceptors protein expression. The molecular mechanisms leading to myocardial dysfunction observed in diabetic myocardial tissue include an unbalance between the pro-oxidant and antioxidant compounds and increased inflammatory process, in terms of TNF-α plasma levels and NFkB activation. We observed that OB corrects oxidative unbalance and reduces inflammatory processes in diabetic myocardium, although, on the basis of our data, we do not provide a clear evidence that the initial mechanism upon which OB acts is related to a direct antioxidative action. We thus investigated the possible molecular mechanisms and intracellular pathway involved in the protective effect of OB. OB enhanced AMPK phosphorylation and up-regulated pro-survival kinases Akt, ERK1/2 and GSK3β in diabetic myocardial tissue. The ability of OB to enhance pAMPK levels in myocardial cells was confirmed by in both in vivo and in vitro models after acute administration of this peptide. Interestingly, the effects of OB were comparable to those induced by metformin, an anti-diabetic drug known to stimulate AMPK. AMPK has recently emerged as an important intracellular signaling pathway in the heart [31]. In the heart, AMPK is modulated by hormones such as adiponectin, leptin and ghrelin, or cytokines like TNFα. A recent paper by Paiva [41], showing that the administration of metformin during the early reperfusion significantly enhances AMPK phosphorylation, concomitantly with the reduction of infarct size, highlighted the important role of AMPK activation in cardioprotective mechanisms. It has been proposed that AMPK may protect against reperfusion injury by increasing glucose uptake, if the potential negative consequences of increased fatty acid oxidation are not present [42]. Since ATP from glycolysis may be preferentially used to support membrane activities such as ion pumping, a shift in glucose metabolism may play an important role in cardiac contractile function, metabolic activity and calcium homeostasis under conditions of calcium overload, such as post-ischemic recovery [43] or during β-adrenergic stimulation [44].

In addition, AMPK activation has been involved in the reduction of inflammatory markers such as TNFα and NFkB transcription factor [45]. Together with Akt, AMPK is considered the main signaling molecule controlling cardiac functions [46]. The observed decrease of cardiac AMPK phosphorylation may therefore contribute, together with reduced Akt signaling, to the depressed myocardial contractility observed in diabetic rats. Indeed, it has been recently reported that AMPK activation may induce an enhancement of L-type calcium current I(Ca) and a prolongation of the action potential duration in cardiomyocytes [47].

Besides AMPK, OB induced phosphorylation of Akt, ERK1/2 and GSK3β, pro-survival kinases known to exert a protective role against oxidative stress [20,23,48], and proposed as integral components of a protective cascade involved in myocardial preconditioning against I/R [49,50]. The involvement of these kinases in the action of OB has been already shown in the proliferation of human retinal pigment epithelial and gastric cancer cells [51,52], and in rodent β-cells and human pancreatic islets cell survival [10,11].

Oxidative stress can trigger the opening of the mitochondrial membrane permeability transition pore (mPTP) and lead to a significant loss of mitochondrial NAD^+^ stores and subsequent derangement of cellular energy reserves and intact cellular functions. Mitochondrial dysfunction plays an important role in the development of diabetes and insulin resistance, and proper cellular function during diabetes requires the maintenance of mitochondrial membrane potential [23]. The present study shows that OB also promoted phosphorylation/inactivation of GSK3β. GSK3β is a substrate of multiple pro-survival protein kinases, including Akt and ERK1/2, and is therefore a step to which multiple protective signaling pathways converge [53]. Since phosphorylated GSK3β limits the opening of mPTP, it is reasonable to think that GSK3β inactivation plays a crucial role in the protective effect afforded by OB in diabetic myocardial tissue.

We cannot exclude, however, that the protective effect of OB may result from additional mechanisms not investigated in the present study, such as the nitric oxide (NO) pathway activation. Recent results, indeed, indicate that increased NO availability attenuates some alterations in metabolism and gene expression associated with insulin resistance induced by a high fat diet [54].

A still open question regards the earliest receptor-initiated mechanisms involved in the action of OB. Although OB was initially claimed to activate the G protein-coupled receptor-39 (GPR39) [4], subsequent studies were unable to demonstrate binding of OB to GPR39 or a stimulatory function of the OB peptide on GPR39-transfected cells [8,9], and the original proposal that OB acts as a ligand of GPR39 has been retracted [28]. We have recently provided evidence that specific OB receptors are present in ventricular myocardial cells [18], with a receptor density and binding affinity quite close to those previously found in rodent and human pancreatic β-cells, where OB promotes cell survival and induces expression of genes involved in the regulation of β-cell mass and function [10,11]. In conclusion, although to date the precise role of OB in cardiovascular pathophysiology remains still partially unknown, the observed beneficial effect in diabetic myocardial tissue confirms the relevance of this peptide as a physiological agent exerting protective effects against cardiac dysfunction and oxidative stress, already shown in the case of ischemic/reperfused heart.

## Competing interests

No conflict of interest, financial or otherwise, are declared by the authors.

## Authors’ contribution

Authors’ contribution: experiments planning and manuscript drafting: MA, GA and GM; cell culture and animal treatment: CG, EA and RM; cardiac contractility studies: EB and GA; biochemical parameters detection, western blot and PCR: MA and RM. All author read and approved the final manuscript.

## Funding

This research was supported by Turin University funding (ex-60%), CRT Foundation 2010, RF 2010.1954 Turin Italy (MA) and Istituto Nazionale per le Ricerche Cardiovascolari (INRC), Bologna, Italy (GA).
